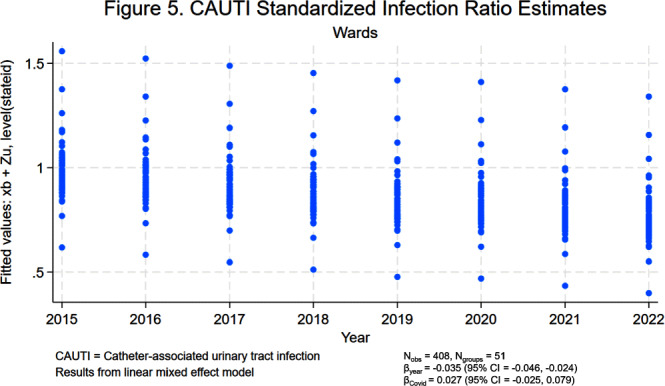# Infection prevention making a difference on statewide standardized infection ratios for device-associated HAIs from 2015-2022

**DOI:** 10.1017/ash.2024.134

**Published:** 2024-09-16

**Authors:** Sai Pranathi Bingi, Stephanie Stroever

**Affiliations:** TTUHSC School of Medicine; Texas Tech University Health Sciences Center

## Abstract

**Background:** Central line-associated bloodstream infections (CLABSIs) and catheter-associated urinary tract infections (CAUTIs) cause significant morbidity and mortality among hospitalized patients. Over the last 10 to 20 years, hospital accountability for the prevention of device-associated infections increased at the state and national levels. For example, the Centers for Medicare & Medicaid Services implemented the Hospital Inpatient Quality Reporting Program in 2015. The objective of this study was to assess the impact of increased federal attention on infection prevention using longitudinal data from the National Healthcare Safety Network (NHSN). We hypothesize that there was a significant decrease in statewide standardized infection ratios (SIRs) for CLABSI and CAUTI over the last 8 years. **Methods:** We collected SIRs for CLABSI and CAUTI in acute care hospitals for all 50 states and Washington D.C. from the NHSN database from 2015 to 2022. For CLABSI, we performed unique analyses for critical care units, wards, and neonatal intensive care (NICU) locations. For CAUTI, we stratified by critical care units and wards. We included all states with more than 5 hospitals reporting data. Those with fewer than 5 were excluded by listwise deletion in the corresponding analysis. We tested trends over time using linear mixed effect models with year as fixed effect and state as random effect. We also included an indicator variable representing the influence of SARS-CoV-2 (Covid-19) on healthcare-associated infections (HAIs). We elected an alpha of 0.05 as the threshold for statistical significance. **Results:** Overall, CLABSI and CAUTI SIRs exhibited significant negative slopes (Figures 1-5) after controlling for the influence of Covid-19. Each analysis revealed progressively lower SIRs compared to the previous year except for the 2019-2020 interval. Interestingly, the linear trend resumed after 2020 with subsequently lower SIRs in 2021 and 2022. Covid-19 had a greater influence on CLABSI SIRs in critical care settings compared to ward or NICU locations. The slope of CAUTI SIRs were impacted less by Covid-19 in wards compared to critical care settings. **Conclusion:** The results of the analysis demonstrate that CLABSI and CAUTI are trending in the desired direction despite the HAI spike during Covid-19. Government and hospital stakeholders in the United States should be encouraged by the reported trends and continue to prioritize the funding and use of resources for evidence-based device-associated infection prevention.

**Figure:**
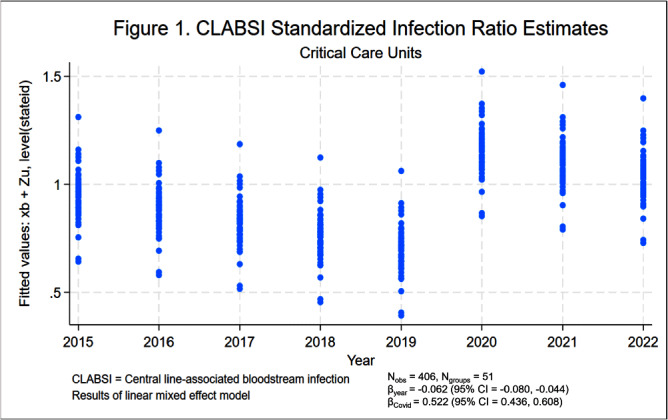

**Figure:**
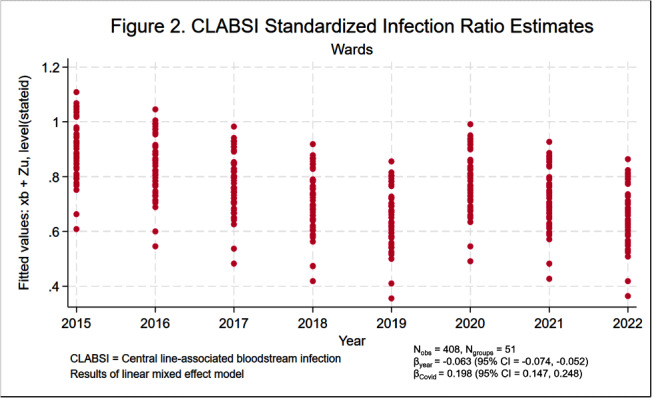

**Figure:**
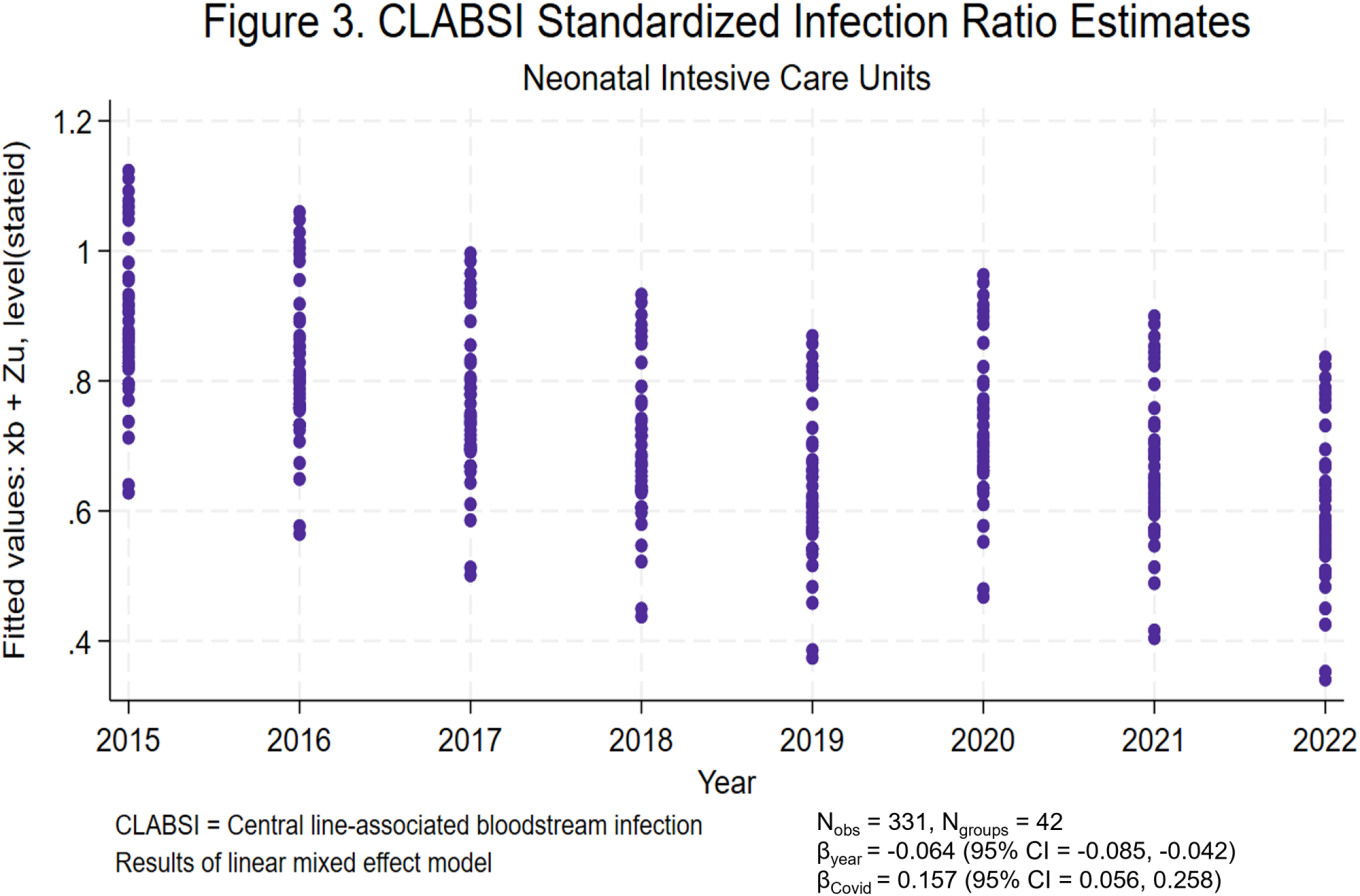

**Figure:**
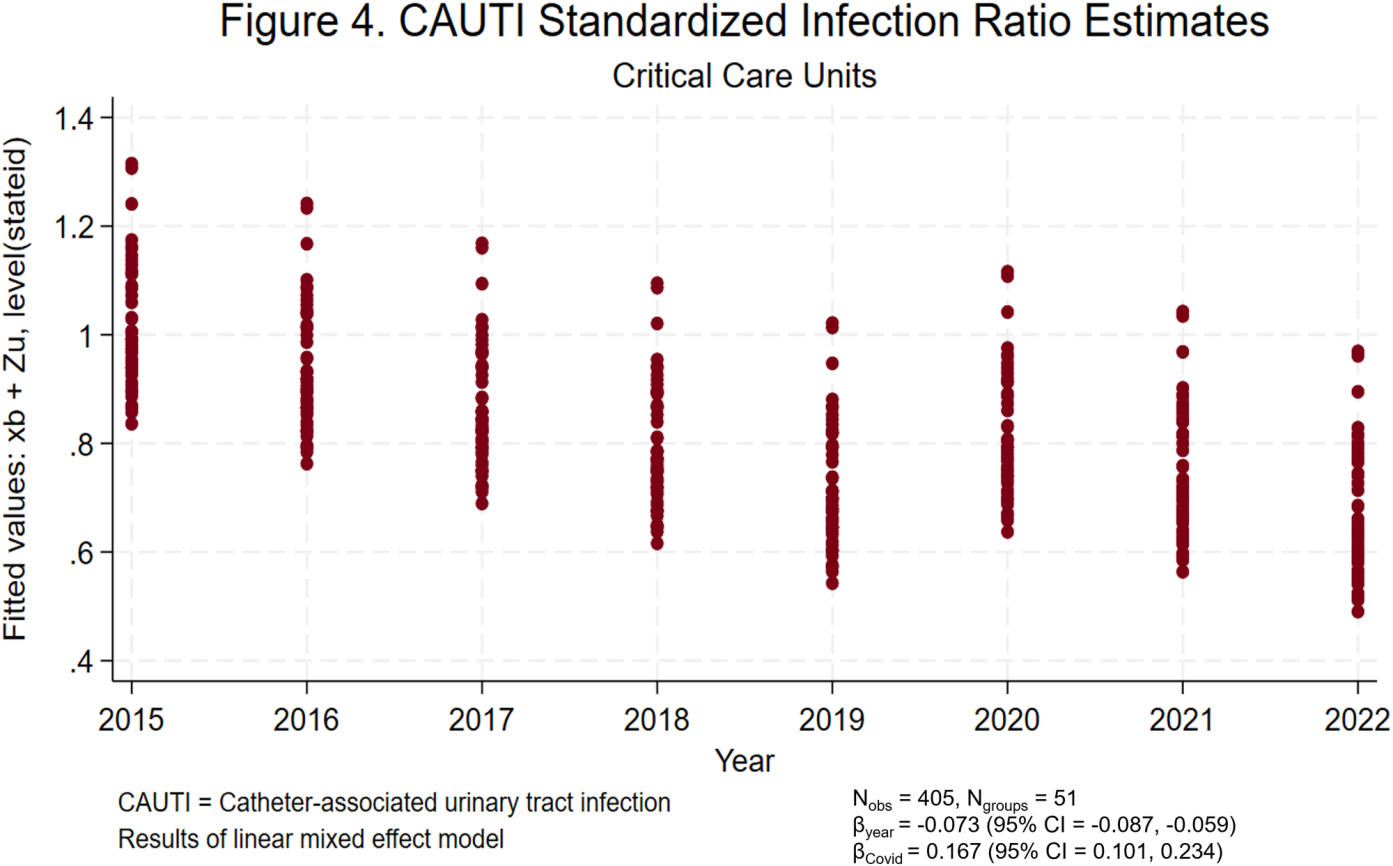

**Figure:**